# Proteomic analysis of lymphoblastoid cell lines from schizophrenic patients

**DOI:** 10.1038/s41398-019-0461-2

**Published:** 2019-04-22

**Authors:** Akira Yoshimi, Shinnosuke Yamada, Shohko Kunimoto, Branko Aleksic, Akihiro Hirakawa, Mitsuki Ohashi, Yurie Matsumoto, Kazuhiro Hada, Norimichi Itoh, Yuko Arioka, Hiroki Kimura, Itaru Kushima, Yukako Nakamura, Tomoko Shiino, Daisuke Mori, Satoshi Tanaka, Shuko Hamada, Yukihiro Noda, Taku Nagai, Kiyofumi Yamada, Norio Ozaki

**Affiliations:** 1 Division of Clinical Sciences and Neuropsychopharmacology, Faculty and Graduate School of Pharmacy, Meijo University, Nagoya, 468-8503 Japan; 2 Department of Neuropsychopharmacology and Hospital Pharmacy, Nagoya University Graduate School of Medicine, Nagoya, 466-8550 Japan; 3 Department of Psychiatry, Nagoya University Graduate School of Medicine, Nagoya, 466-8550 Japan; 4 Department of Biostatistics and Bioinformatics, Graduate School of Medicine, University of Tokyo, Tokyo, 113-0033 Japan; 5 Center for Advanced Medicine and Clinical Research, Nagoya University Hospital, Nagoya, 466-8550 Japan; 6 Department of Psychiatry, Nagoya University Hospital, Nagoya, 466-8550 Japan; 7 Institute for Advanced Research, Nagoya University, Nagoya, 464-8601 Japan; 8 Department of Pathology of Mental Diseases, National Institute of Mental Health, National Center of Neurology and Psychiatry, Tokyo, 187-8553 Japan; 9 Brain and Mind Research Center, Nagoya University, Nagoya, 466-8550 Japan

**Keywords:** Predictive markers, Diagnostic markers

## Abstract

Although a number of studies have identified several convincing candidate genes or molecules, the pathophysiology of schizophrenia (SCZ) has not been completely elucidated. Therapeutic optimization based on pathophysiology should be performed as early as possible to improve functional outcomes and prognosis; to detect useful biomarkers for SCZ, which reflect pathophysiology and can be utilized for timely diagnosis and effective therapy. To explore biomarkers for SCZ, we employed fluorescence two-dimensional differential gel electrophoresis (2D-DIGE) of lymphoblastoid cell lines (LCLs) [1st sample set: 30 SCZ and 30 control subjects (CON)]. Differentially expressed protein spots were sequenced by liquid chromatography tandem-mass spectrometry (LC-MS/MS) and identified proteins were confirmed by western blotting (WB) (1st and 2nd sample set: 60 SCZ and 60 CON). Multivariate logistic regression analysis was performed to identify an optimal combination of biomarkers to create a prediction model for SCZ. Twenty protein spots were differentially expressed between SCZ and CON in 2D-DIGE analysis and 22 unique proteins were identified by LC-MS/MS. Differential expression of eight of 22 proteins was confirmed by WB. Among the eight candidate proteins (HSPA4L, MX1, GLRX3, UROD, MAPRE1, TBCB, IGHM, and GART), we successfully constructed logistic regression models comprised of 4- and 6-markers with good discriminative ability between SCZ and CON. In both WB and gene expression analysis of LCL, MX1 showed reproducibly significant associations. Moreover, *Mx1* and its related proinflamatory genes (*Mx2*, *Il1b*, and *Tnf*) were also up-regulated in poly I:C-treated mice. Differentially expressed proteins might be associated with molecular pathophysiology of SCZ, including dysregulation of immunological reactions and potentially provide diagnostic and prognostic biomarkers.

## Introduction

Schizophrenia (SCZ) is a chronic and disabling mental disorder, typically occurring after puberty, with a lifetime prevalence of ~1% in the global population. Symptoms of SCZ are characterized by various clinical features including positive (psychosis, hallucinations, delusions, and disorganized speech/thinking/behaviour) and negative (apathy, lack of emotion, and social withdrawal) symptoms, as well as cognitive deficits (difficulties in working and long-term memory, attention, and executive function), which cause enormous personal and societal burdens^1^. The duration of untreated psychosis has been reported as one of the most relevant clinical response predictors for SCZ. Untreated psychosis results in changes in brain structure and function due to neurotoxic effects, and treatment delays worsen clinical and social outcomes^2^. However, since there is no objective biomarker for SCZ, it is difficult to provide more precise early-stage detection. Therefore, reliable biomarkers based on the pathophysiology of SCZ have been desired and could point towards new therapeutic/preventive strategies^3^.

SCZ is a disease of the central nervous system (CNS) and investigations of brain samples will no doubt provide meaningful information, however it is very difficult to obtain brain biopsy samples from patients. Although post-mortem brains have been investigated, these samples are affected by various confounding factors related to the age at death, medication, cause of death, agonal state, post-mortem interval, and brain pH^4^. Several studies have suggested that CNS alterations might be reflected in peripheral tissues based on gene expression profiles between brain and blood^5^. Although fresh blood is useful for screening peripheral biomarkers, it is also affected by various confounding factors such as health condition, diet, medication, smoking, and circadian rhythms^3^. In contrast, the use of cell-lines with repeated passaging could reduce the effect of the above mentioned confounding factors and is useful to identify molecules underlying pathophysiology^6^. Proteomic approaches, as well as microarray or next generation sequencing (NGS)-based transcriptome studies, have been demonstrated to be particularly useful for screening molecular expression changes to obtain novel insights into disease^7^. In particular, proteomic analyses have many advantages related to the major role of proteins in molecular function, thereby reflecting disease state and/or traits. Therefore, proteomics could provide molecular biology based diagnostic tools (biomarkers) for monitoring symptom severity, treatment responses, and predicting progression.

To explore differentially expressed proteins and potential biomarkers for SCZ, we conducted two-dimensional fluorescence difference gel electrophoresis (2D-DIGE) analyses of lymphoblastoid cell lines (LCLs).

## Materials and methods

### Subjects

Two independent sample groups were used in this study (Supplementary Table S1). All subjects were unrelated to each other and ethnically Japanese. The SCZ diagnosis was made by at least two experienced psychiatrists and was based on unstructured patient interviews and reviews of their medical records in accordance with the Diagnostic and Statistical Manual of Mental Disorders, Fourth Edition, Text Revision (DSM-IV-TR) criteria. All control subjects (CON) were selected from the general population and psychiatrically screened by interviews. This study was approved by the Ethics Review Committee of Nagoya University Graduate School of Medicine and Nagoya University Hospital (No. 1033), and was conducted in accordance with the Helsinki Declaration. Written informed consent was obtained from each subject.

### Cell cultures

LCLs derived from SCZ (*n* = 60) and CON (*n* = 60) subjects were established with the widely-used Epstein-Barr virus (EBV) transformation^8^, with minor modifications. In brief, 5 mL of venous blood was drawn into a vacuum blood collection tube with sodium heparin, and lymphocytes ware isolated using Ficoll-Paque (Amersham Biosciences, Piscataway, NJ, USA) density gradient centrifugation. After washing the lymphocytes with saline, cells were cultured in Roswell Park Memorial Institute (RPMI) 1640 medium with 2 mM L-glutamine and 5 mg/L phenol red (Gibco, Big Cabin, OK, USA), supplemented with 20% heat-inactivated foetal bovine serum (FBS, single lot, as was media to minimize variation; Gibco), 8 mg/L tylosin tartrate (Sigma-Aldrich, St. Louis, MO, USA), 50 U/mL penicillin-50 μg/mL streptomycin (Gibco), filtered supernatant of B95–8 cell cultures infected by EBV (VR-1492; American Type Culture Collection, Rockville, MD, USA), and 2 µg/mL cyclosporine A (CyA) (Sandimmune; Novartis Pharma, Tokyo, Japan) under optimal growth conditions at 37 °C in a humidified 5% CO_2_ incubator. Cells were passaged twice a week using RPMI1640 medium (supplemented with 10–20% heat-inactivated FBS, 8 µg/mL tyrosine tartrate, 50 U/mL penicillin-50 μg/mL streptomycin without EBV and CyA). After colony formation, cells were pelleted and stored in liquid nitrogen until analyses. For expression analyses, stored LCLs were re-cultured by rapidly thawing at 37 °C and were grown in RPMI1640 medium at 37 °C with 5% CO_2_. Cell growth was monitored with a haemocytometer, and cells were cultured at a density of ~3.0 × 10^5^ cells/mL by exchanging the medium with fresh media twice a week. To avoid the cause of altered cellular function (e.g. enhancement of endoplasmic reticulum stress response)^9^, we used LCLs with the number of freeze and thaw only at once. In addition, to minimize differences in expression profiles due to culture and sample workup conditions, all samples were cultured and harvested at the same time using the same media preparation and extraction reagents.

### 2D-DIGE analysis

To explore differentially expressed protein profiles between SCZ and CON, LCLs from the 1st sample set (SCZ *n* = 30, CON *n* = 30) were homogenized in lysis buffer (20 mM Tris-HCl, pH 7.5, 2 mM EDTA, 0.5 mM EGTA, 1 mM dithiothreitol (DTT), 1% Triton X-100, protease inhibitor cocktail, phosphatase inhibitor cocktail) and centrifuged at 16000 × *g* for 20 min at 4 °C. The soluble supernatant was purified and concentrated with methanol/acetone precipitation and reconstituted in resuspension buffer (30 mM Tris–HCl, pH 8.5, 4% CHAPS, 7 M urea, 2 M thiourea). Protein concentrations were determined using a commercial protein assay kit (Bio-Rad, Hercules, CA, USA).

2D-DIGE was carried out according to a previously published method^10^ with minor modifications. An equal amount of protein (30 µg) from each LCL was individually labelled using 240 pmol of either Cy3 or Cy5 from a CyDye DIGE Fluor Minimal Labelling Kit (GE Healthcare, Chalfont St. Giles, Buckinghamshire, UK), according to the manufacturer’s protocol. For spot normalization to allow comparison across different gels, we prepared an internal standard (IS) protein pool consisting of equal amounts of all samples, which was labelled with Cy2. Fluorescently labelled proteins were diluted with an equal volume of sample buffer [40 mM DTT, 4% CHAPS, 7 M urea, 2 M thiourea, 1% pharmalyte (broad range pH 3–10)]. Different kinds of fluorescent-labelled LCL protein samples from SCZ, CON, and IS were mixed before loading on the gel. Mixed samples were added to a final volume (450 µL) of rehydration buffer [20 mM DTT, 4% CHAPS, 7 M urea, 2 M thiourea, 0.5% pharmalyte, 0.001% bromophenol blue (BPB)] and were applied to IPG gel strips with a separation range of pH 3–10 (24 cm Immobiline DryStrip pH 3–10 NL, 240 × 3 × 0.5 mm; GE Healthcare). After 12 h of rehydration at 20 °C, isoelectric focusing (IEF) was carried out as follows; initially at 30 V for 2 h, at 100 V for 1 h, at 200 V for 5 min, and then gradually increasing the voltage to 8 000 V for 8.5 h, and finally maintaining at 8 000 V until reaching 60,000 Vh in an Ettan IPGphor 3 Isoelectric Focusing System (GE Healthcare), maintaining a limiting current of 50 µA per strip at 20 °C.

After IEF separation, the drystrip gels were equilibrated for 25 min in sodium dodecyl sulphate (SDS)-equilibration buffer (50 mM Tris-HCl, pH 8.8, 6 M urea, 30% glycerol, 2% SDS) with 1% DTT for reduction. Equilibration was repeated in the SDS-equilibration buffer for another 10 min with 2.5% iodoacetamide for alkylation. The second-dimensional separation was carried out on a 10% SDS-polyacrylamide gel (24 × 20 × 0.1 cm). SDS-polyacrylamide gel electrophoresis (SDS-PAGE) was performed using a two-step protocol; at 30 °C, 10 mA/gel, 80 V, 1 W/gel for an hour, and then 12 mA/gel, 150 V, 2 W/gel for 15–17 h until the loading marker reached the edge of the gel in the Ettan DALT*six* Large Electrophoresis System (GE Healthcare).

Fluorescence dye (Cy2, Cy3, and Cy5) labelled proteins were visualized by scanning gels at 100 µm resolution using a Typhoon Trio laser scanner (GE Healthcare). A total of 87 images from 29 gels with good separation quality were analysed using PDQuest 2-D Analysis Advanced Software Version 8.0 (Bio-Rad). The abundance of each Cy3- or Cy5-labeled protein spot was normalized according to the corresponding protein spot from the Cy2-labeled IS sample.

### Mass spectrometry

For protein identification, 100 µg of the un-labelled IS protein pool was separated by 2D-PAGE as described above. The proteins were visualized by silver staining and the protein spots of interest were excised. The gel pieces including proteins were destained in destaining solution (0.2% K_3_Fe(CN)_6_ and 0.02% Na_2_S_2_O_3_) for 15 min, washed in Milli-Q water for 10 min, dehydrated in CH_3_CN, and dried in a centrifuge-vacuum dryer (Spin Dryer Lite VC-36R; TAITEC) for 15 min. The gels were rehydrated and reduced with 100 mM DTT in 100 mM NH_4_HCO_3_ for 30 min at 50 °C, and then dehydrated as described above. The gel pieces were alkylated with 100 mM iodoacetamide in 100 mM NH_4_HCO_3_ for 30 min at room temperature (RT), dehydrated, and rehydrated by 20 µg/mL trypsin (Promega Benelux, Leiden, The Netherlands) in 100 mM NH_4_HCO_3_ for 30 min on ice. A solution of 100 mM NH_4_HCO_3_ (10–15 μL) was added, and the proteins were digested overnight at 37 °C. Peptides were extracted three times with 50 μL of Solution A (2% CH_3_CN and 0.1% TFA), 33 µL of Solution B (98% CH_3_CN and 0.1% TFA), and 42 µL of Solution B. The extracts were dried and dissolved in Solution A (total 10–12 µL) prior to application to the sample vial. Each sample of peptides was loaded on a Paradigm MS4 HPLC system (Michrom BioResources, Auburn, CA, USA) equipped with a magic C18AQ column of 0.1 mm in diameter and 50 mm in length (Michrom BioResources). Reversed-phase chromatography was performed with a linear gradient of solvent A (2% CH_3_CN in 0.1% HCOOH) and solvent B (90% CH_3_CN in 0.1% HCOOH), 5% solvent B at 0 min to 100% solvent B at 45 min with a 1 μL/min flow rate. Ionization was performed using an ADVANCE CaptiveSpray Source (Michrom BioResources) with a capillary voltage of 1.7 kV and temperature of 150 °C. A precursor ion scan was carried out using a 400–2000 mass to charge ratio (m/z) prior to MS/MS analysis with an LCQ advantage mass spectrometer (Thermo Fisher Scientific, Waltham, MA, USA). Multiple peptide mass fingerprinting and MS/MS peptide fragmentation data were used for searching the Swiss-Prot protein database with the MASCOT program (Matrix Science, Boston, MA, USA) for MS/MS ion searching with 2.0 D mass tolerance. Identified proteins were classified according to functional ontology using the Human Protein Reference Database (HPRD: http://www.hprd.org).

### Western blotting

Equal amounts (15 µg) of proteins extracted from LCLs of the 1st (SCZ *n* = 30, CON *n* = 30) and 2nd (SCZ *n* = 30, CON *n* = 30) sample sets (see above procedure) were diluted in sample buffer (final concentration: 62.5 mM Tris-HCl, pH 6.8, containing 2% SDS, 10% glycerol, 0.5% BPB, and 5% 2-mercaptoethanol) and denatured at 95 °C for 5 min. Proteins were separated by SDS-PAGE using 5% stacking gels and 8–12% running gels and transferred to PVDF membranes using a Trans-Blot SD Semi-Dry Transfer Cell (Bio-Rad). Membrane blots were processed using the SNAP i.d. protein detection system (Millipore), according to the manufacturer’s protocol, or placing the blots into a Hybri-Bag at RT for 1 h or overnight at 4 °C, with specific antibodies or horseradish peroxidase-conjugated secondary antibodies (Supplementary Table S2). The positive bands were revealed using enhanced chemiluminescence substrate for western blotting (WB) reagents (PerkinElmer, Waltham, MA, USA) and visualized with an ImageQuant LAS 4000mini (GE Healthcare). The intensities of the bands were quantified using ImageQuant TL 7.0 software (GE Healthcare).

### Animals

ICR mice were obtained from Japan SLC Inc. (Hamamatsu, Japan) and maintained under standard specific pathogen-free environmental conditions. Pregnant mice were monitored for the parturition date, which was taken as postnatal day (PD) 0. They were housed under a standard 12-h light/dark cycle (lights on at 9:00) at a constant temperature of 23 ± 1 ℃, with free access to food and water throughout the experiments. We used male mice exclusively to minimize any potential variability due to sex-specific effects. The animals were handled in accordance with the guidelines established by the Institutional Animal Care and Use Committee of Nagoya University Graduate School of Medicine (approval ID: 30316, 30416) and Meijo University Faculty of Pharmacy (approval ID: 2018-P-E-16), the Guiding Principles for the Care and Use of Laboratory Animals approved by the Japanese Pharmacological Society, and the National Institutes of Health Guide for the Care and Use of Laboratory Animals (NIH Publications No. 8023, revised 1978).

### Gene expression analysis of prefrontal cortex and hippocampus of poly I:C-treated mice

All litters were randomly divided into saline- or poly I:C-treated groups. From PD 2 to 6, mice were injected s.c. daily with either pyrogen-free saline or poly I:C (Sigma–Aldrich) at a dose of 5 mg/kg^11^. Neonatal mice were decapitated 2 or 24 h after the final treatment with saline or poly I:C and their brains were removed. Total RNA was isolated using the RNeasy Plus Mini Kit (Qiagen, Hilden, Germany) from each mouse (2 h after the final treatment with saline *n* = 6 or poly I:C *n* = 6, 24 h after the final treatment with saline *n* = 6 or poly I:C *n* = 6). Total RNA isolated from the prefrontal cortex (PFC) and hippocampus (HIP) was converted into cDNA using a high capacity RNA-to-cDNA Kit (Applied Biosystems, Foster City, CA, USA). Levels of mRNA expression were quantified using an ABI Prism 7900HT Real-Time PCR System (Applied Biosystems) with the KAPA SYBR^®^ FAST qPCR Kit (Kapa Biosystems, Boston, MA, USA), according to the manufacturer’s directions. The primer pairs for eight candidate markers confirmed by WB (Table 2), proinflammatory mediators (Myxovirus resistance (Mx) 2: *Mx1*; Interferon α: *Ifna*; Interferon β: *Ifnb*; Interferon γ: *Ifng*; Interleukin 1β: *Il1b*; Interleukin 6: *Il6*; Tumor necrosis factor α: *Tnf*; Nuclear factor of kappa light polypeptide gene enhancer in B cells 1, p105: *Nfkb*), and a housekeeping gene (β-actin: *Actb*) are described in Supplementary Table S3.

### Statistical analysis

Sample sizes were determined based on literature^10,12^ and were not calculated using statistical methods. Data collection and analysis were not blind to the sample conditions. Data were analyzed using either Student’s *t*-test or Welch’s *t*-test for comparisons between two groups. The comparison of expression levels of identified candidate markers was performed using univariate logistic regression analysis. To search optimal prediction models for SCZ, multivariate logistic regression approach was performed. *P* values less than 0.05 were considered to be statistically significant.

## Results

### Altered protein expression in LCLs between SCZ and CON

A representative merged 2D-DIGE image of LCL from SCZ and CON subjects is shown in Supplementary Figure S1. A total of 1 174 protein spots were identified on typical LCL gels [SCZ 1 102.8 (s.d. = 13.3); CON 1 102.6 (s.d. = 16.2)], with 20 spots being differentially expressed between SCZ-LCL and CON-LCL, of which seven spots were increased while 13 spots were decreased in SCZ-LCL (Table 1). These 20 differentially expressed protein spots were selected for protein identification by liquid chromatography tandem-mass spectrometry (LC-MS/MS) and 22 unique proteins were successfully identified with high MASCOT scores (Table 1).

**Table 1:** Identification of differentially expressed protein spots in 2D-DIGE analysis

Spot No.	Protein name	Gene symbol	Gene map locus	Accession No.	Functional ontlogy^a^			2D-DIGE^b^		LC-MS/MS	
					Molecular class	Molecular function	Biological process	FC^c^	*P*-value^d^	MASCOT score	Theoretical *pI*/*m*^e^
1	Kinesin-like protein KIF11	*KIF11*	*10q24.1*	P52732	Motor protein	Motor activity	Cell growth/maintenance	1.20	0.004	93	5.47/119,158.99
2	Heat shock 70 kDa protein 4 L	*HSPA4L*	*4q28*	O95757	Heat shock protein	Heat shock protein activity	Protein metabolism	0.78	0.039	393	5.63/94,512.49
3	Heat shock protein HSP 90-beta	*HSP90AB1*	*6p12*	Q6PK50	Chaperone	Chaperone activity	Cell communication; Signal transduction	0.91	0.043	177	4.89/40,295.18
	Pyridoxal-dependent decarboxylase domain-containing protein 1	*PDXDC1*	*16p13.11*	Q86XE2	Enzyme: decarboxylase	Carboxy-lyase activity	Metabolism; Energy pathways			155	6.21/54,982.42
4	Vacuolar protein sorting-associated protein 35	*VPS35*	*16q12*	Q96QK1	Transport/cargo protein	Transporter activity	Transport	1.73	0.026	814	5.32/91,707.03
5	Interferon-induced GTP-binding protein Mx1	*MX1*	*21q22.3*	P20591	GTPase	GTPase activity	Cell communication; Signal transduction	1.13	0.050	1545	5.60/75,520.34
6	Interferon-induced GTP-binding protein Mx1	*MX1*	*21q22.3*	P20591	GTPase	GTPase activity	Cell communication; Signal transduction	1.14	0.009	1616	5.60/75,520.34
7	Interferon-induced GTP-binding protein Mx1	*MX1*	*21q22.3*	P20591	GTPase	GTPase activity	Cell communication; Signal transduction	1.20	0.018	1202	5.60/75,520.34
8	Plastin-2	*LCP1*	*13q14.3*	P13796	Calcium binding protein	Calcium ion binding	Cell communication; Signal transduction	0.86	0.046	1315	5.29/70,288.39
9	Histidyl-tRNA synthetase, cytoplasmic	*HARS*	*5q31.3*	P12081	Enzyme: ligase	Ligase activity	Protein metabolism	0.94	0.045	1322	5.72/57,410.51
10	Glutaredoxin-3	*GLRX3*	*10q26*	O76003	Unclassified	Molecular function unknown	Biological process unknown	0.92	0.040	446	5.31/37,432.03
11	Uroporphyrinogen decarboxylase	*UROD*	*1p34*	P06132	Enzyme: decarboxylase	Carboxy-lyase activity	Metabolism; Energy pathways	0.84	0.049	135	5.77/40,786.91
12	Inorganic pyrophosphatase	*PPA1*	*10q11.1-24*	Q15181	Enzyme: phosphohydrolase	Catalytic activity	Metabolism; Energy pathways	0.83	0.025	505	5.54/32,660.04
13	Annexin A5	*ANXA5*	*4q26-q28; 4q28-32*	P08758	Calcium binding protein	Calcium ion binding	Cell communication; Signal transduction	0.91	0.023	527	4.93/35,936.77
14	Microtubule-associated protein RP/EB family member 1	*MAPRE1*	*20q11.1-11.23*	Q15691	Cell cycle control protein	Protein binding	Cell communication; Signal transduction	0.87	0.003	238	5.02/29,999.08
	EF-hand domain-containing protein D2	*EFHD2*	*1p36.21*	Q96C19	Unclassified	Molecular function unknown	Biological process unknown			254	5.15/26,697.28
	Tubulin-folding cofactor B	*TBCB*	*19q13.11-13.12*	Q99426	Chaperone	Chaperone activity	Protein metabolism			244	5.06/27,325.53
15	Ubiquitin carboxyl-terminal hydrolase isozyme L1	*UCHL1*	*4p14*	P09936	Ubiquitin proteasome system protein	Ubiquitin-specific protease activity	Protein metabolism	0.53	0.013	164	5.33/24,824.34
16	Adenine phosphoribosyltransferase	*APRT*	*16q24*	P07741	Enzyme: ribosyltransferase	Transferase activity	Purine salvage	0.92	0.015	375	5.75/19,607.77
17	Ig mu chain C region	*IGHM*	*14q32.33*	P01871	Immunogloblin	Antigen binding	Immune response	1.48	0.031	496	6.35/49,306.59
18	Trifunctional purine biosynthetic protein adenosine-3	*GART*	*21q22.1; 21q22.11*	P22102	Enzyme: transferase	Ligase activity	Regulation of nucleobase, nucleoside, nucleotide and nucleic acid metabolism	0.93	0.042	509	6.26/107,767.19
19	Elongation factor 1-gamma	*EEF1G*	*11q12.3*	P26641	Translation regulatory protein	Translation regulator activity	Protein metabolism	1.18	0.047	248	6.25/50,118.81
20	Beta-lactamase-like protein 2	*LACTB2*	*8q22-22.3*	Q53H82	Unclassified	Molecular function unknown	Biological process unknown	0.88	0.039	221	6.32/32,805.65
	Putative deoxyribonuclease TATDN1	*TATDN1*	*8q24.13*	Q6P1N9	Unclassified	Molecular function unknown	Biological process unknown			100	6.51/33,601.66

*FC* fold change

^a^Functional ontology: proteins were classified according to functional ontology using the Human Protein Reference Database (HPRD: http://www.hprd.org)

^b^2D-DIGE: evaluation and normalization of protein spot intensities and statistical test were performed by PDQuest software

^c^FC = the ratio of the average intensity (SCZ/CON)

^d^*P*-value: the differentially expressed protein spots were determined by Student’s *t*-test

^e^Theoretical isoelectric point (*pI*) and molecular mass (*m*) according to the sequence

### Western blotting validation

WB analysis of the 22 identified proteins was performed to confirm the results of the 2D-DIGE analysis in 1st sample set (SCZ *n* = 30, CON *n* = 30). The direction of change was confirmed in eight of the 22 proteins (Table 2, Supplementary Figures S2 and S3). MX1 and IGHM were significantly up-regulated in SCZ-LCL, while HSPA4L, GLRX3, UROD, MAPRE1, TBCB, and GART were significantly down-regulated in the 1st sample set. These eight proteins were examined for further validation analysis using an independent 2nd sample set (SCZ *n* = 30, CON *n* = 30). As a result, three proteins (HSPA4L, MX1, and GART) were found to be differentially expressed in the 2nd sample set with the same direction of change (Table 2 and Supplementary Figure S4). When we combined the results of the two sample sets, four proteins (HSPA4L, MX1, TBCB, and GART) were found to be significantly altered (Table 2 and Supplementary Figure S5).

**Table 2:** Results of Western blotting analysis of LCLs

Spot No.	Candidate marker	1st sample set (CON *n* = 30, SCZ *n* = 30)						2nd sample set (CON *n* = 30, SCZ *n* = 30)						Combinede (CON *n* = 60, SCZ *n* *=* 60)					
		FC^a^	*t*-test *P*-value^b^		Logistic regression analysis			FC	*t*-test *P*-value		Logistic regression analysis			FC	*t*-test *P*-value		Logistic regression analysis		
					OR	*P*-value	AUC				OR	*P*-value	AUC				OR	*P*-value	AUC
1	KIF11	1.31	0.284	(S)	2.10	0.289	0.63												
2	HSPA4L	0.62	0.017	(W)	0.50	0.029	0.64	0.73	0.015	(W)	0.05	0.021	0.65	0.65	<0.001	(W)	0.59	0.03	0.61
3	HSP90AB1	1.12	0.390	(S)	1.45	0.384	0.57												
	PDXDC1	1.10	0.604	(S)	1.15	0.598	0.49												
4	VPS35	0.88	0.295	(S)	0.39	0.296	0.55												
5, 6, 7	MX1	1.39	0.006	(S)	9.26	0.011	0.72	1.32	0.006	(W)	12.76	0.012	0.68	1.35	<0.001	(W)	10.32	<0.001	0.70
8	LCP1	1.04	0.791	(S)	1.07	0.787	0.54												
9	HARS	1.09	0.588	(S)	1.55	0.582	0.57												
10	GLRX3	0.74	0.002	(W)	0.05	0.006	0.73	1.12	0.103	(S)	13.31	0.106	0.61	0.88	0.073	(S)	0.33	0.08	0.55
11	UROD	0.77	0.010	(S)	0.24	0.018	0.73	1.03	0.824	(W)	1.17	0.828	0.54	0.85	0.118	(S)	0.60	0.12	0.59
12	PPA1	0.99	0.953	(S)	0.97	0.952	0.50												
13	ANXA5	0.88	0.105	(W)	0.32	0.109	0.61												
14	MAPRE1	0.76	0.009	(S)	0.20	0.015	0.69	1.18	0.010	(S)	95.47	0.015	0.68	0.89	0.182	(S)	0.57	0.19	0.51
	EFHD2	0.99	0.908	(S)	0.95	0.906	0.47												
	TBCB	0.68	0.024	(W)	0.20	0.034	0.66	0.95	0.628	(S)	0.68	0.622	0.57	0.82	0.035	(S)	0.36	0.04	0.60
15	UCHL1	0.97	0.919	(S)	0.99	0.917	0.54												
16	APRT	0.59	0.248	(W)	0.90	0.356	0.49												
17	IGHM	1.80	0.037	(W)	5.03	0.050	0.63	0.71	0.336	(W)	0.98	0.336	0.49	0.80	0.466	(S)	0.98	0.46	0.45
18	GART	0.75	0.022	(S)	0.15	0.031	0.66	0.73	0.002	(S)	0.02	0.005	0.74	0.74	<0.001	(W)	0.08	<0.001	0.70
19	EEF1G	1.16	0.154	(S)	2.35	0.158	0.65												
20	LACTB2	1.10	0.358	(S)	1.76	0.356	0.56												
	TATDN1	1.14	0.195	(W)	2.58	0.210	0.57												

*FC* fold change, *OR* odds ratio (SCZ/CON), *AUC* the area under the receiver operating characteristic curves, *Combined* combined results of 1st sample set and 2nd sample set

^a^FC = the ratio of the average intensity (SCZ/CON)

^b^*t*-test *P*-value: the differentially expressed proteins were determined by Student’s *t*-test (S) or Welch’s *t*-test (W) (unpaired, two tailed)

### Statistical prediction model

Among the eight promising biomarkers validated by WB in the 1st sample set, optimal 4-marker model (comprised of MX1, GLRX3, UROD, and GART; equation 1) and 6-maker model (comprised of MX1, GLRX3, UROD, MAPRE1, TBCB, and GART; equation 2) were constructed by multivariate logistic regression analysis.

1 $$p = \frac{{\exp (6.44 - 3.82GART - 2.18UROD + 2.48MX1 - 2.84GLRX3)}}{{1 + \exp (6.44 - 3.82GART - 2.18UROD + 2.48MX1 - 2.84GLRX3)}}$$

2 $$p = \frac{{\exp (11.54 - 4.64GART - 2.67UROD + 2.04MX1 - 3.14GLRX3 - 1.65MARPE1 - 2.01TBCB)}}{{1 + \exp (11.54 - 4.64GART - 2.67UROD + 2.04MX1 - 3.14GLRX3 - 1.65MARPRE1 - 2.01TBCB)}}$$

MX1 and GART showed reproducibly significant associations in both the 4- and 6-marker models (Table 3). The area under the receiver operating characteristic curves (AUCs) for the accuracy of predicting SCZ using the 4- and 6-marker models were 0.86 and 0.88, respectively (Table 3 and Supplementary Figure S6), implying good discriminative ability. For external validation of these statistical prediction models, the WB results using the 2nd sample set were applied to the 4- and 6-marker models. The AUCs of 4- and 6-marker models were 0.72 and 0.66, respectively (Table 3). The predicting accuracies of two most prominently altered markers, MX1 and GART, showed high performance even only applying as a single protein marker (AUC_MX1_: 0.68–0.72 and AUC_GART_: 0.66–0.74; Table 2).

**Table 3:** Prediction accuracy of 4- and 6-marker models

Variable	1st sample set (CON *n* = 30, SCZ *n* = 30)			2nd sample set (CON *n* = 30, SCZ *n* = 30)			Combined (CON *n* = 60, SCZ *n* = 60)		
	OR	*P*-value^a^	Prediction accuracy^b^	OR	*P*-value	Prediction accuracy	OR	*P*-value	Prediction accuracy
*4-marker model*									
MX1	11.91	0.017		26.57	0.016		19.46	<0.001	
GLRX3	0.06	0.036	81.7%	4.99	0.507	73.3%	0.46	0.384	77.5%
UROD	0.11	0.011	(AUC = 0.86)	0.55	0.609	(AUC = 0.72)	0.44	0.108	(AUC = 0.82)
GART	0.02	0.006		0.01	0.006		0.01	<0.001	
*6-marker model*									
MX1	7.68	0.079		16.72	0.049		20.48	<0.001	
GLRX3	0.04	0.03		2.37	0.741		0.48	0.431	
UROD	0.07	0.012	81.7%	0.79	0.875	78.3%	0.51	0.235	77.5%
GART	0.01	0.011	(AUC = 0.88)	0.003	0.008	(AUC = 0.66)	0.01	<0.001	(AUC = 0.82)
MAPRE1	0.19	0.095		103.8	0.052		0.73	0.629	
TBCB	0.13	0.050		1.93	0.616		0.42	0.138	

*OR* odds ratio, *Combined* combined results of 1st sample set and 2nd sample set, *AUC* the area under the receiver operating characteristic curves

^a^*P*-value: multivariate logistic regression analysis

^b^Prediction accuracy: [1—overall misclassification rate (OMR)] × 100 (%)

### Gene expression analysis of prefrontal cortex and hippocampus of poly I:C-treated mice

Since the most promising markers for SCZ identified from LCL experiments were related to the immunological reactions, we analysed the mRNA levels of candidate markers and proinflammatory mediators in PFC and HIP of poly I:C-treated mice, which is an animal neurodevelopmental model of SCZ. Among the genes examined, mRNA levels of *Mx1* (PFC/HIP), *Mx2* (PFC/HIP), *Il1b* (HIP), and *Tnf* (HIP) were significantly increased 2 h after the final treatment of poly I:C, compared to the saline-treated control group (Table 4). At 24 h after poly I:C treatment, *Ighm* (PFC/HIP) was increased, while *Mapre1* (HIP), *Ifnb* (HIP), and *Il6* (PFC) were decreased in the poly I:C-treated group. Although mRNA level of *Gart* was not changed in both PFC and HIP at 2 and 24 h after poly I:C treatment (Table 4), repeated poly I:C treatment in neonatal mice are likely cause an inflammatory response in PFC and HIP.

**Table 4:** Results of quantitative real-time PCR analysis of poly I:C-treated mice

Gene symbol	2 h^a^				24 h^b^			
	PFC		HIP		PFC		HIP	
	FC^c^	*P*-value^d^	FC	*P*-value	FC	*P*-value	FC	*P*-value
*Candidate marker*								
*Hspa4l*	1.39	0.176	0.63	0.292	1.07	0.425	1.00	0.994
*Mx1*	2.53	0.039	1.80	0.043	1.03	0.804	0.85	0.243
*Glrx3*	1.07	0.266	0.99	0.763	1.19	0.109	1.13	0.126
*Urod*	0.97	0.754	0.90	0.171	1.13	0.091	1.13	0.259
*Mapre1*	1.02	0.913	0.95	0.458	1.05	0.758	0.82	0.022
*Tbcb*	0.97	0.879	0.86	0.390	1.01	0.964	0.96	0.676
*Ighm*	1.22	0.156	1.20	0.054	1.19	0.009	1.23	0.024
*Gart*	1.63	0.139	1.11	0.222	0.98	0.699	0.93	0.330
*Proinflammatory mediator*								
*Mx2*	1.53	0.029	1.61	0.042	1.13	0.310	0.96	0.528
*Ifna*	1.35	0.347	1.26	0.265	0.87	0.439	0.67	0.069
*Ifnb*	1.44	0.280	1.21	0.392	0.87	0.391	0.64	0.039
*Ifng*	1.56	0.070	1.52	0.063	1.09	0.647	1.22	0.169
*Il1b*	1.34	0.171	1.52	0.014	0.98	0.854	1.30	0.076
*Il6*	1.03	0.931	1.24	0.432	0.62	0.013	0.62	0.089
*Tnf*	1.60	0.108	2.49	0.005	0.74	0.189	0.72	0.116
*Nfkb1*	0.94	0.377	0.90	0.278	1.00	0.990	0.99	0.972

*PFC* prefrontal cortex, *HIP* hippocampus, *FC* fold change

^a^2 h: sacrificed 2 h after final treatment with saline (*n* = 6) or polyI:C (*n* = 6)

^b^24 h: sacrificed 24 h after final treatment with saline (*n* = 6) or polyI:C (*n* = 6)

^c^FC = the ratio of the average intensity (poly I:C/saline)

^d^*P*-value: the differentially expressed proteins were determined by Student’s *t*-test

## Discussion

### Main findings

In the present study, we conducted a comparative proteomic analysis of LCL proteins derived from SCZ and CON subjects (Supplementary Table S1). Twenty-two unique proteins were identified as differentially expressed markers for SCZ using 2D-DIGE and LC-MS/MS (Table 1). Eight proteins (up-regulated in SCZ-LCL: MX1 and IGHM; down-regulated in SCZ-LCL: HSPA4L, GLRX3, UROD, MAPRE1, TBCB, and GART) were confirmed after WB validation using the 1st sample set (Table 2 and Supplementary Figure S3). External validation confirmed that HSPA4L, MX1, and GART were differentially expressed between SCZ-LCL and CON-LCL with the same direction, while MAPRE1 was not consistent with the 1st sample set (Table 2 and Supplementary Figure S4). Overall, HSPA4L, MX1, TBCB, and GART were significantly altered in the combined analysis of the two sample sets (Table 2 and Supplementary Figure S5), suggesting that these proteins were potentially related to dysfunctional molecular pathways in SCZ. Interestingly, the gene expression level of *MX1* in LCL and that of *Mx1* in the brain (prefrontal cortex and hippocampus) of poly I:C-treated mice (one of the most widely used animal model for schizophrenia^11^) were also significantly elevated (Supplementary Table S4 and Table 4); therefore MX1 might be an important molecule for understanding SCZ pathophysiology.

Among the eight candidate proteins (HSPA4L, MX1, GLRX3, UROD, MAPRE1, TBCB, IGHM, and GART) confirmed in the 1st sample set (Table 2 and Supplementary Figure S4), we tried to construct a statistical prediction model for SCZ using multivariate logistic regression analysis, and two combinations of proteins (4- and 6-marker models) proved suitable for discriminating SCZ and CON with high performance (Table 3 and Supplementary Figure S6). According to Occam’s razor, a simpler model is preferred over a more complex model. Increasing the number of variables leads to a more complicated model and may result in over fitting. In order to avoid this problem, the model with fewer variables is considered to be the better statistical model. Consistent with this concept, the prediction accuracy of the two models was found to be equivalent. Therefore, the 4-marker model is a good prediction model with better accuracy. Furthermore, statistical model validation using independent samples indicated that MX1 and GART showed reproducibly significant associations in both the 4- and 6-marker models (Table 3). These proteins were also strongly associated in the WB validation and also showed high performance for discriminating SCZ and CON in univariate logistic regression analysis (Table 2), suggesting these proteins might be promising biomarkers for SCZ.

### Possible mechanisms

Functional characterization of the identified molecules indicated that they were involved in metabolism, energy pathways, and signal transduction (Table 1). Impairments in these pathways were previously identified as a SCZ trait by genetic studies, gene and protein expression studies, and functional analyses^13–15^, suggesting that the proteins identified in this study may be potentially involved in SCZ pathophysiology.

MX1, the most promising molecular signature for SCZ, is a type I interferon (IFN)-inducible protein that exhibits antiviral activity against a variety of RNA viruses^16^. The reported antiviral activity of MX1 indicates that it accelerates cell death through caspase-dependent/independent mechanisms and enhances ER-stress signalling^16^. Epidemiological studies have shown that maternal virus infection during pregnancy increases the risk of SCZ in the offspring^17^ and animal models based on the prenatal infection hypothesis of SCZ, including prenatal exposure to human influenza virus^18^, polyinosinic-polycytidylic acid (poly I:C)^19^, lipopolysaccharide (LPS)^20^, turpentine^21^, and interleukin (IL)-6^22^, have been observed to produce behavioural and cognitive dysfunctions. It was suggested that the neuropathological outcomes might be affected by immunological reactions associated with proinflammatory mediators such as IFNs, IL-1β, IL-6, TNF-α, and nitric oxide (NO), which eventually cause neurotoxic effects^23^. Interestingly, our preliminary experiments using poly I:C model mice showed the increase of *Mx1* gene expression as well *Il1b* and *Tnf* (Table 4), suggesting that up-regulation of *Mx1* might reflect the dysregulation of immunological reactions associated with the pathophysiology of SCZ.

GART, another promising marker, has phosphoribosylglycinamide formyltransferase, phosphoribosylglycinamide synthetase, phosphoribosylaminoimidazole synthetase activity, and promotes *de novo* purine biosynthesis^24^. Extracellular purines can activate astrocytes and microglial cells in response to CNS injury or neurodegeneration and increased levels of purine nucleotides may promote astrocytic hypertrophy^25^. Zhang et al. (2014)^26^ reported that GART is mainly localized in astrocytes and its expression is enhanced after CNS injury and neuroinflammation, which eventually causes astrocyte activation and neural apoptosis. Activated astrocytes release many different types of potentially neurotoxic and proinflammatory mediators, including cytokines and growth factors such as IFNs, IL-1β, IL-6, TNF-α, innate immunity mediators such as LPS and Toll-like receptor (TLR) ligands, neurotransmitters such as glutamate and noradrenalin, purine nucleotides such as adenosine triphosphate (ATP), and reactive oxygen species (ROS) such as NO^27^. Thus, GART might be involved in the activation of astrocytes and regulate proinflammatory mediator-related immunological reactions^26^. Although poly I:C-treated mice did not show the changes in mRNA level of *Gart* in both PFC and HIP (Table 4), the most promising molecules might be involved in the pathophysiology of SCZ. Further investigation should be performed to elucidate molecular functions of candidate molecules identified in this study.

### Limitation and future research

A couple of limitations should be considered in the present study. First, although 2D-DIGE technique have improved dynamic range, several issues related to reproducibility due to the properties of proteins (e.g. low abundance, high acidity/basicity, extreme size, or high hydrophobicity). To detect low abundance proteins and maximize discovery rate, we applied high-sensitive spot detection parameters and no multiple testing correction. Although we performed visual inspection of the gel images in 2D-DIGE and Western blotting verification of all candidates from differentially expressed protein spots, appropriate data mining procedures to reduce false discovery should have be considered. Second, the prediction model constructed from eight candidates showed reproducibly high discrimination performance, however, there were inconsistencies of the protein expression change between two sample cohorts of LCLs. The verification of reproducibility using large sample sets should be conducted. Third, it is necessary to consider the utility of different sample types, such as plasma, serum, and peripheral blood mononuclear cells. Different samples analysed using a variety of methodologies have the potential to enhance the understanding of biological processes related to SCZ^28^.

Differentially expressed protein markers, identified from LCLs, might reflect pathophysiology of disease. Recently available biological resources (e.g. induced pluripotent stem cells and induced microglia-like cells) and genome editing tools (e.g. CRISPER/Cas, TALEN) are undoubtedly powerful technologies for enabling functional analyses of CNS resources such as cell lines or organoids from living donors^29,30^. Future follow-up studies, which investigate molecular mechanisms of identified markers, could provide insight into the pathophysiology of SCZ and potentially provide novel molecular targets and diagnostic/prognostic biomarkers.

## Supplementary information

Supplementary Information
