# Author Correction: Proteomic analysis of lymphoblastoid cell lines from schizophrenic patients

**DOI:** 10.1038/s41398-019-0479-5

**Published:** 2019-05-03

**Authors:** Akira Yoshimi, Shinnosuke Yamada, Shohko Kunimoto, Branko Aleksic, Akihiro Hirakawa, Mitsuki Ohashi, Yurie Matsumoto, Kazuhiro Hada, Norimichi Itoh, Yuko Arioka, Hiroki Kimura, Itaru Kushima, Yukako Nakamura, Tomoko Shiino, Daisuke Mori, Satoshi Tanaka, Shuko Hamada, Yukihiro Noda, Taku Nagai, Kiyofumi Yamada, Norio Ozaki

**Affiliations:** 1 Division of Clinical Sciences and Neuropsychopharmacology, Faculty and Graduate School of Pharmacy, Meijo University, Nagoya, 468-8503 Japan; 2 Department of Neuropsychopharmacology and Hospital Pharmacy, Nagoya University Graduate School of Medicine, Nagoya, 466-8550 Japan; 3 Department of Psychiatry, Nagoya University Graduate School of Medicine, Nagoya, 466-8550 Japan; 4 Department of Biostatistics and Bioinformatics, Graduate School of Medicine, University of Tokyo, Tokyo, 113-0033 Japan; 5 Center for Advanced Medicine and Clinical Research, Nagoya University Hospital, Nagoya, 466-8550 Japan; 6 Department of Psychiatry, Nagoya University Hospital, Nagoya, 466-8550 Japan; 7 Institute for Advanced Research, Nagoya University, Nagoya, 464-8601 Japan; 8 Department of Pathology of Mental Diseases, National Institute of Mental Health, National Center of Neurology and Psychiatry, Tokyo, 187-8553 Japan; 9 Brain and Mind Research Center, Nagoya University, Nagoya, 466-8550 Japan

**Correction to: Translational Psychiatry**

10.1038/s41398-019-0461-2 Published online 22 April 2019

The original Article required a few updates; one co-author name, which was given as Hiroki Kiumura, has been updated to Hiroki Kimura. Furthermore, supplementary information has been updated, and grant numbers have been added. These updates have been made to both the PDF and HTML versions of this Article.

