# Supplementary material for: Proteomic analysis of lymphoblastoid cell lines from schizophrenic patients
Source: Transl Psychiatry. 2019 Apr 22;9:126. doi: 10.1038/s41398-019-0461-2 (PMC6476876; doi:10.1038/s41398-019-0461-2)
Supplement: Supplementary file 1 — Supplementary Information [file 41398_2019_461_MOESM1_ESM.doc]

**Proteomic Analysis of Lymphoblastoid Cell Lines from Schizophrenic Patients**

Akira Yoshimi, Ph.D.^1,2,3¶^, Shinnosuke Yamada, Ph.D.^1,2,¶^, Shohko Kunimoto, Ph.D.^3,¶^, Branko Aleksic, M.D., Ph.D.^3,*^, Akihiro Hirakawa, Ph.D.^4^, Mitsuki Ohashi^1^, Yurie Matsumoto^1,3^, Kazuhiro Hada^2^, Norimichi Itoh, Ph.D.^2^, Yuko Arioka, Ph.D.^3,5^, Hiroki Kimura, M.D., Ph.D.^3,6^, Itaru Kushima, M.D., Ph.D.^3,6,7^, Yukako Nakamura, Ph.D.^3^, Tomoko Shiino, Ph.D.^3,8^, Daisuke Mori, Ph.D.^3,9^, Satoshi Tanaka, M.D., Ph.D.^6^, Shuko Hamada, Ph.D.^3^, Yukihiro Noda, Ph.D.^1,2,3^, Taku Nagai, Ph.D.^2^, Kiyofumi Yamada, Ph.D.^2^ and Norio Ozaki, M.D., Ph.D.^3,6^

^1^Division of Clinical Sciences and Neuropsychopharmacology, Faculty and Graduate School of Pharmacy, Meijo University, Nagoya 468-8503, Japan; ^2^Department of Neuropsychopharmacology and Hospital Pharmacy, Nagoya University Graduate School of Medicine, Nagoya 466-8550, Japan; ^3^Department of Psychiatry, Nagoya University Graduate School of Medicine, Nagoya 466-8550, Japan; ^4^Department of Biostatistics and Bioinformatics, Graduate School of Medicine, University of Tokyo, 113-0033, Japan; ^5^Center for Advanced Medicine and Clinical Research, Nagoya University Hospital, Nagoya 466-8550, Japan; ^6^Department of Psychiatry, Nagoya University Hospital, Nagoya 466-8550, Japan; ^7^Institute for Advanced Research, Nagoya University, Nagoya 464-8601, Japan; ^8^Department of Pathology of Mental Diseases, National Institute of Mental Health, National Center of Neurology and Psychiatry, Tokyo 187-8553, Japan

^9^Brain and Mind Research Center, Nagoya University, Nagoya 466-8550, Japan

^¶^These authors contributed equally to this work.

**^*^Corresponding author:**

**Contents of Supplementary Information**

I. Establishment of lymphoblastoid cell lines

II. Gene expression analysis of lymphoblastoid cell line

III. Supplementary Table S1. Sample information.

IV. Supplementary Table S2. Western blotting conditions.

V. Supplementary Table S3. Primer sequences for quantitative real-time PCR analysis.

VI. Supplementary Table S4. Results of quantitative real-time PCR analysis of lymphoblastoid cell line.

VII. Supplementary Figure S1. 2D-DIGE images of LCLs.

VIII. Supplementary Figure S2. Western blotting images of LCLs.

IX. Supplementary Figure S3. Results of Western blotting analysis of 1^st^ sample set of LCLs.

X. Supplementary Figure S4. Results of Western blotting analysis of 2^nd^ sample set of LCLs.

XI. Supplementary Figure S5. Results of Western blotting analysis of 1^st^ and 2^nd^ sample sets of LCLs.

XII. Supplementary Figure S6. ROC curves of 4- and 6-marker models for predicting schizophrenia.

XIII. Reference

**I. Establishment of lymphoblastoid cell lines**

Lymphoblastoid cell lines (LCLs) derived from patients with schizophrenia (SCZ) and healthy control subjects (CON) (Supplementary Table S1) were established with the widely used Epstein-Barr virus (EBV) transformation [^1^](#_ENREF_1) with minor modifications. In brief, 5 mL of venous blood was drawn into a vacuum blood collection tube with sodium heparin, and lymphocytes ware isolated using Ficoll-Paque (Amersham Biosciences, Piscataway, NJ, USA) density gradient centrifugation. After washing the lymphocytes with saline, cells were cultured in Roswell Park Memorial Institute (RPMI) 1640 medium with 2 mM L-glutamine and 5 mg/L phenol red (Gibco, Big Cabin, OK, USA), supplemented with 20% heat-inactivated foetal bovine serum (FBS, single lot, as was media to minimize variation; Gibco), 8 mg/L tylosin tartrate (Sigma-Aldrich, St. Louis, MO, USA), 50 U/mL penicillin-50 μg/mL streptomycin (Gibco), filtered supernatant of B95-8 cell cultures infected by EBV (VR-1492; American Type Culture Collection, Rockville, MD, USA), and 2 µg/mL cyclosporine A (CyA) (Sandimmune; Novartis Pharma, Tokyo, Japan) under optimal growth conditions at 37°C in a humidified 5% CO_2_ incubator. Cells were passaged twice a week using RPMI1640 medium (supplemented with 10-20% heat-inactivated FBS, 8 µg/mL tyrosine tartrate, 50 U/mL penicillin-50 μg/mL streptomycin without EBV and CyA). After colony formation, cells were pelleted and stored in liquid nitrogen until analyses.

**II. Gene expression analysis of lymphoblastoid cell line**

Methods: For gene expression analysis, total RNA was extracted from LCLs using an RNAqueous Kit (Ambion, Austin, TX, USA) and treated with DNase to remove contaminated genomic DNA using a TURBO DNA-free Kit (Ambion), then reverse transcribed to complementary DNA (cDNA) with a high capacity RNA-to-cDNA Kit (Applied Biosystems, Foster City, CA, USA). Concentration, A260/A280, and A260/A230 values for RNA and cDNA samples were obtained using a NanoDrop 8000 Spectrophotometer (Thermo Fisher Scientific). Two housekeeping genes, beta-2-microglobulin (*B2M*) and glucuronidase-beta (*GUSB*), were selected as internal control genes to normalize the polymerase chain reaction (PCR) data. Quantitative real-time PCR (qPCR) was performed on an ABI Prism 7900HT Real-Time PCR System (Applied Biosystems) using predesigned TaqMan Gene Expression Assay probes (Hs00204675_m1 for *HSPA4L*, Hs00895608_m1 for *MX1*, Hs01582641_g1 for *GLRX3*, Hs01099754_g1 for *UROD*, Hs01121102_g1 for *MAPRE1*, Hs00998434_m1 for *TBCB*, Hs00941538_g1 for *IGHM*, Hs00894582_m1 for *GART*, Hs99999907_m1 for *B2M* and Hs99999908_ml for *GUSB*; Applied Biosystems). Measurement of cycle thresholds was performed in duplicate. The data, including amplification efficiency and relative expression on quantification, were analysed using the comparative cycle threshold (Ct) method. Comparisons of expression levels were performed using the unpaired Student’s t-test.

Results: The expression of the genes encoding the differentially expressed proteins confirmed by WB from the 1^st^ sample set (Table 2 and Supplementary Figure S4) was also investigated in the SCZ-LCL and CON-LCL samples. Only *GLRX3* was down-regulated in the 1^st^ sample set, while *UROD* and *MAPRE1* were up-regulated in the 2^nd^ sample set (Supplementary Table S4). When we combined the 1^st^ and 2^nd^ sample sets, *MX1*, *UROD*, and *MAPRE1* were up-regulated and *GLRX3* was down-regulated (Supplementary Table S4).

**III. Supplementary Table S1. Sample information.**

**IV. Supplementary Table S2. Western blotting conditions.**

**V. Supplementary Table S3. Primer sequences for quantitative real-time PCR analysis.**

**VI. Supplementary Table S4. Results of quantitative real-time PCR analysis of lymphoblastoid cell line.**

**VII. Supplementary Figure S1. 2D-DIGE images of LCLs.**


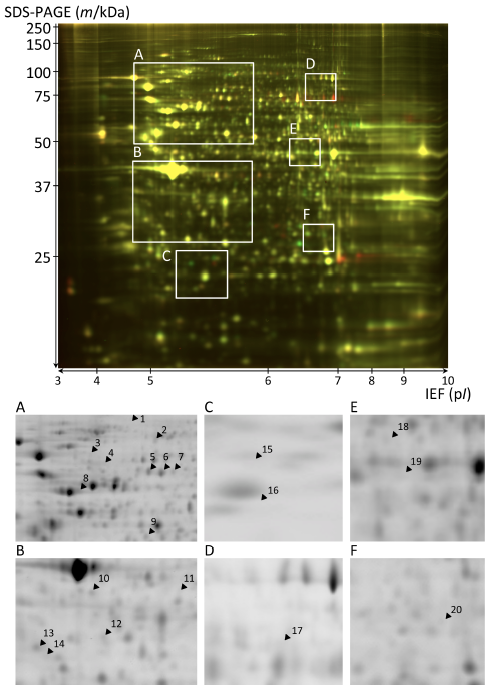
A representative merged 2D-DIGE image of CyDye-labelled proteins derived from SCZ-LCL (red) and CON-LCL (green). The boxes (A to F) show areas including differentially expressed protein spots and the arrowheads indicate the protein spots of interest with their spot numbers (see Tables 1 and 2).

**VIII. Supplementary Figure S2. Western blotting images of LCLs.**

A typical example of Western blotting images of 22 target proteins identified by 2D-DIGE.

**IX. Supplementary Figure S3. Results of Western blotting analysis of 1^st^ sample set of LCLs.**

**X. Supplementary Figure S4. Results of Western blotting analysis of 2^nd^ sample set of LCLs.**

The bar chart shows the relative expression of each target protein by Western blotting. Each value was calculated as the ratio of normalized signal intensity by glyceraldehyde 3-phosphate dehydrogenase (GAPDH), compared to that of CON. LCL: lymphoblastoid cell line, CON: control *n* = 30, SCZ: schizophrenia *n* = 30. Bars represent the mean ± s.e.m. *P < 0.05, **P < 0.01 (Student’s t-test, unpaired, two tailed), ^†^P <0.05, ^††^P < 0.01 (Welch’s t-test, unpaired, two tailed).

**XI. Supplementary Figure S5. Results of Western blotting analysis of 1^st^ and 2^nd^ sample sets of LCLs.**

The bar chart shows the relative expression of each target protein by Western blotting. Each value was calculated as the ratio of normalized signal intensity by glyceraldehyde 3-phosphate dehydrogenase (GAPDH), compared to that of CON. LCL: lymphoblastoid cell line, CON: control *n* = 60, SCZ: schizophrenia *n* = 60. Bars represent the mean ± s.e.m. *P < 0.05 (Student’s t-test, unpaired, two tailed), ^†††^P < 0.001 (Welch’s t-test, unpaired, two tailed).

**XII. Supplementary Figure S6. ROC curves of 4- and 6-marker models for predicting schizophrenia.**

ROC curves of (a) 4-marker models and (b) 6-marker models were drawn for the 1^st^ sample set (blue), 2^nd^ sample set (green), and combined (1^st^ and 2^nd^ sample set; red).

**XIII. Reference**

1. Kato T., et al. Mechanisms of altered Ca2+ signalling in transformed lymphoblastoid cells from patients with bipolar disorder*. Int. J. Neuropsychopharmaco*l**.** 6, 379-389 (2003).
